# Intense, carrier frequency and bandwidth tunable quasi single-cycle pulses from an organic emitter covering the Terahertz frequency gap

**DOI:** 10.1038/srep14394

**Published:** 2015-09-24

**Authors:** C. Vicario, B. Monoszlai, M. Jazbinsek, S. -H. Lee, O. -P. Kwon, C. P. Hauri

**Affiliations:** 1 Paul Scherrer Institute, SwissFEL, 5232 Villigen PSI, Switzerland; 2 Rainbow Photonics AG, 8048 Zurich, Switzerland; 3 Department of Molecular Science and Technology, Ajou University, Suwon 443-749, Korea; 4 Ecole Polytechnique Fédérale de Lausanne, 1015 Lausanne, Switzerland

## Abstract

In Terahertz (THz) science, one of the long-standing challenges has been the formation of spectrally dense, single-cycle pulses with tunable duration and spectrum across the frequency range of 0.1–15 THz (*THz gap*). This frequency band, lying between the electronically and optically accessible spectra hosts important molecular fingerprints and collective modes which cannot be fully controlled by present strong-field THz sources. We present a method that provides powerful single-cycle THz pulses in the THz gap with a stable absolute phase whose duration can be continuously selected between 68 fs and 1100 fs. The loss-free and chirp-free technique is based on optical rectification of a wavelength-tunable pump pulse in the organic emitter HMQ-TMS that allows for tuning of the spectral bandwidth from 1 to more than 7 octaves over the entire THz gap. The presented source tunability of the temporal carrier frequency and spectrum expands the scope of spectrally dense THz sources to time-resolved nonlinear THz spectroscopy in the entire THz gap. This opens new opportunities towards ultrafast coherent control over matter and light.

Coherent and broadband radiation in the Terahertz range (T-rays) between 0.1 and 15 THz, the so-called THz gap (hν ≈ 0.4–60 meV), offers exceptional opportunities in life science and fundamental research as it enables new schemes for controlling and sensing matter and light. Different to optical light (hν ≈ 2 eV), which is routinely used to prepare highly excited non-equilibrium states, Terahertz radiation allows direct and selective access to low-energy modes in matter, such as molecular rotations, phonons, polaritons, charge and spin waves etc. by resonant excitation^1^,^2^. The T-ray electromagnetic field cycle is about three orders of magnitude slower than for optical light and therefore matches the natural timescale of those collective modes. The recent advent of stronger T-ray sources permits now even non-resonant (impulsive) and nonlinear control over matter and light^3^,^4^,^5^,^6^.

The source requirements for impulsive applications differ significantly from resonantly-driven experiments. Indeed, the ideal source provides an intense THz pulse with a chirp-free field oscillation at highest possible magnitude, with a carrier field period that is tunable. Such a source is essential for excitation of a non-resonant process at its ultimate speed limit. While high field THz pulses have become recently available, their tunability in carrier frequency and bandwidth is still in its infancy. State of the art intense T-ray sources provide pulse energies of tens of μJ and are based on optical rectification (OR). Unfortunately their emission is typically confined to low THz frequencies (ν < 5 THz)^7^,^8^,^9^,^10^ and bandwidth tunability is limited^11^. In principle, plasma-based THz sources offer broadband emission (0.1–100 THz^12^,^13^,^14^,^15^,^16^,^17^,^18^) but defocusing and other nonlinear effects limits the T-ray energy to a few μJ. Finally we mention that difference frequency generation provides radiation at infrared wavelengths (i.e. ν ≈ 15–100 THz, sometimes also called multi-THz range)^19^ which is, however, outside the targeted THz window.

Here we demonstrate a new concept for high-field single-cycle T-ray pulse generation in the THz gap, which provides a widely tunable carrier oscillation frequency. Our scheme is based on OR in the recently developed HMQ-TMS (2-(4-hydroxy-3-methoxystyryl)-1-methyl-quinolinium-2,4,6-trimethylbenzene-sulfo-nate) organic crystal. The pulses carry a high spectral density across the frequency window of 0.1–15 THz, with the spectral bandwidth that can be varied between 1 to 7 octaves. The loss-free spectral tuning of bandwidth and carrier frequency is based on adjusting the pump laser’s wavelength. This simple approach represents an important advance over modern laser and accelerator based high-field T-ray sources as it offers chirp-free mono-cycle THz pulses adjustable between 68 fs and 1100 fs (i.e. carrier frequency between 0.8 and 15 THz). To our best knowledge such extensive tunability in pulse duration (while keeping the pulse duration at its transform-limit) is unique and not provided by any other T-ray source. This opens novel opportunities in the emerging field of impulsive THz science as it enables a parametric match of the stimulus’ carrier oscillation frequency to the response time of the induced dynamics.

## Results and Discussion

**Figure 1:**
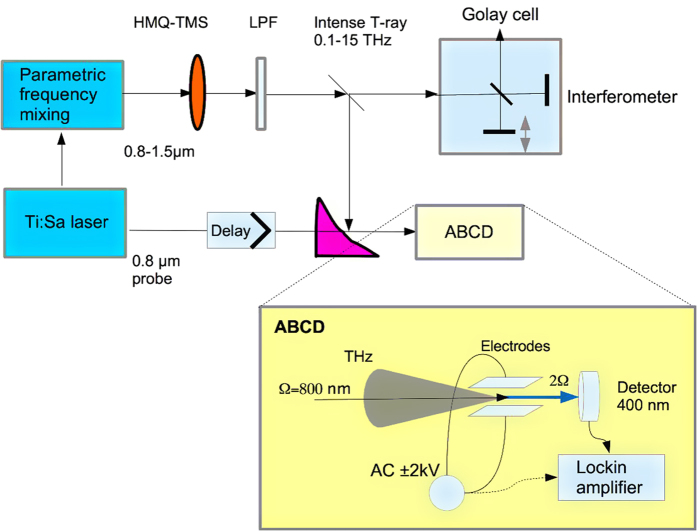
Compact laser-driven Terahertz emitter with shaping capabilities over 7 octaves. The wavelength-tunable output of an optical frequency mixer driven by a powerful Ti:sapphire laser is parametrically rectified in the nonlinear crystal HMQ-TMS, mounted on a glass substrate. Optical rectification gives rise to ultra-intense and ultra-broadband THz radiation which is separated from the pump pulse by a low-pass filter, LPF, at 20 THz (QMC instruments see Supplementary material for the transmission curve). The intense T-ray spectra are measured by means of a Michelson interferometer equipped with a Golay cell. The THz electric field evolution in time is reconstructed with air-biased coherent detector (ABCD). This technique is described in details in the supplementary material. The schematic of this diagnostics is shown in the inset. The THz beam and the probe pulse are both focused in between two high voltage electrodes and generate a second harmonic signal proportional the THz field. The AC bias voltage of amplitude ±2 kV and frequency 25 Hz is used for lockin detection.

Our original table-top Terahertz shaping source is shown in Fig. 1. As driving laser for the optical frequency synthesizer a 20 mJ, 50 fs FWHM pulse from a 100 Hz Ti:sapphire amplifier system is used. The optical frequency synthesizer is based on optical parametric amplifiers and delivers 65 fs (FWHM), multi-mJ pulses tunable in wavelength up to 1.5 μm which are optically rectified in the highly nonlinear crystal HMQ-TMS. The unipolar organic crystal HMQ-TMS^20^ (375 μm thick) is pumped by this wavelength-tunable high-power source with output spectrum covering the near infrared spectral region of 0.8–1.5 μm and pulse duration of 65 fs for all the wavelengths.

**Figure 2:**
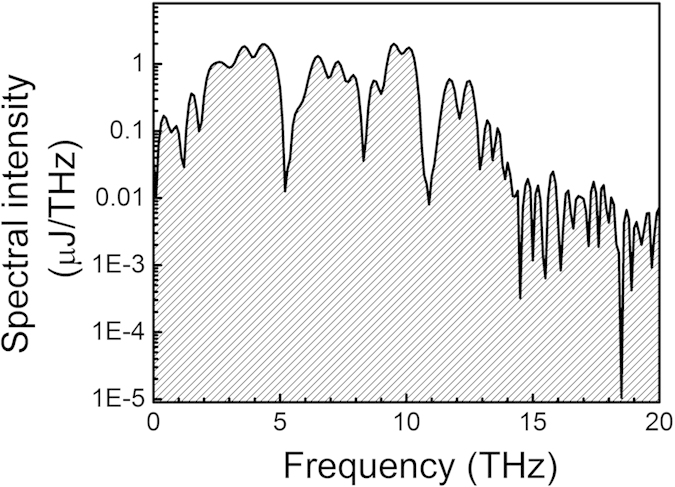
Broadband Terahertz radiation produced in HMQ-TMS retrieved by field autocorrelation. The emission is obtained by optical rectification of 65 fs FWHM laser at 1500 nm central wavelength. The spectrum extends over more than 7 octaves (0.1–15 THz) and fills the previously inaccessible THz gap (0.1–15 THz) at high spectral intensity. The spectral width appears to be limited mainly by the pump pulse duration. The phonon resonances in the HMQ-TMS are responsible for the absorption lines visible in the spectrum.

**Figure 3:**
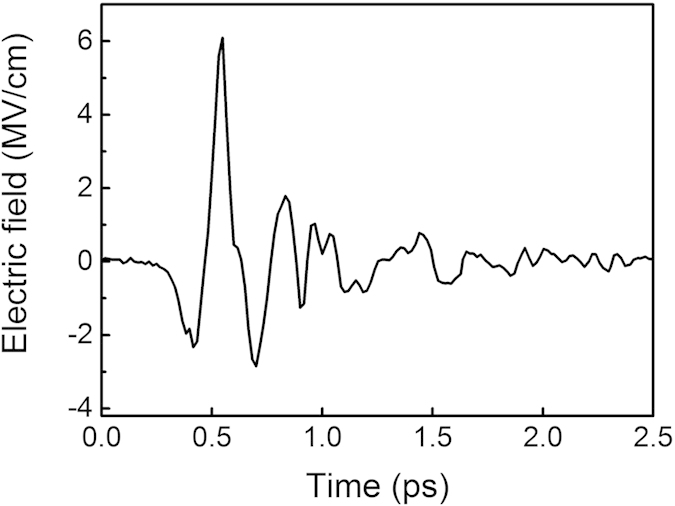
Reconstruction of the electric field using air-biased coherent detection (ABCD) for pump pulse at 1500 nm. The graph clearly indicates a sub-100 fs single cycle pulse, which carries the 7-octave spanning spectrum shown in Fig. 2. The field strength is calculated from the measured energy and focus spot size.

HMQ-TMS shows outstanding properties for THz-wave generation since it combines macroscopic optical nonlinearity as large as DAST crystal (ß_eff_ = 185 × 10^−30^ esu) and extreme phase matching bandwidth due to the optimal molecular packing structure and crystal characteristics^20^. This makes this crystal superior to the conventional inorganic (e.g. ZnTe^21^, LiNbO_3_^22^) and organic^8^,^9^,^10^ Terahertz emitters. Figure 2 shows an example of the exceptionally broad THz emission from HMQ-TMS extending between 0.1 and 20 THz. The radiation is produced by optical rectification of the 1.5 μm pump pulse at fluence of 10 mJ/cm^2^ on the organic crystal. The pump pulse energy of 1.1 mJ was focused on the organic crystal with 0.5 cm^2^ area. The measured continuous THz spectrum covers more than 7 octaves with the highest spectral energy density emitted between 2 and 10 THz at an energy content exceeding 1 μJ/THz. The smallest THz energy content is located in the organic crystal phonon-active absorption lines with intensity of 10 nJ/THz. The corresponding electric field oscillation (Fig. 3) is measured with broad-bandwidth diagnostics based on ABCD (see Supplementary material for further information)^23^. The electric field consists of a prominent single cycle oscillation with extremely short duration (<70 fs). The THz pulse energy is 11 μJ, which results in a peak electric and magnetic field of 6.1 MV/cm and 2 Tesla respectively for a focused spot size of 350 μm FWHM. The THz electric *E*_*THz*_ and magnetic *B*_*THz*_ fields are calculated according to the formula 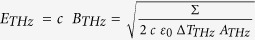, with *c* the speed of light, *ε*_*0*_ the vacuum permittivity, *Σ* the THz energy, *ΔT*_*THz*_ is the pulse duration and *A*_*THz*_ the beam size. The equivalence between THz and optical pump duration is obvious and reflects the observed spectral THz cut-off at approximately 15 THz. Indeed, for larger frequencies the optical rectification process breaks down due to the limited pump spectral width. In contrast to THz sources based on multicolor gas ionization, the optical rectification in HMQ-TMS provides much higher spectral density in the THz gap frequency range. Moreover our approach is easily up-scalable in energy and in THz field by means of higher pump energy and by employing larger crystal size^10^ while keeping the pump fluence at the crystal around 10 mJ/cm^2^. This pump fluence gives maximum OR conversion efficiency and is sufficiently far from nonlinear absorption caused by multiphoton effects and from the optical damage threshold (>20 mJ/cm^2^).

An outstanding feature of the HMQ-TMS crystal is that the optical rectification phase-matching bandwidth depends strongly on the pump wavelength. Therefore the THz spectral bandwidth and consequently the temporal field characteristics (carrier oscillation frequency) of the single-cycle transient can be straightforwardly controlled by the driving laser wavelength. This is an important advance over state of the art shaping techniques. Indeed, spectral control of high-field pulses in the THz gap has been a technological hurdle in the past and strategies for shaping Terahertz pulses have been demonstrated by phase and amplitude shaping of the near-IR driving laser^24^,^25^,^26^,^27^. However, these shaping techniques have been restricted to the low frequency range (<3 THz) and to low THz peak-power.

**Figure 4:**
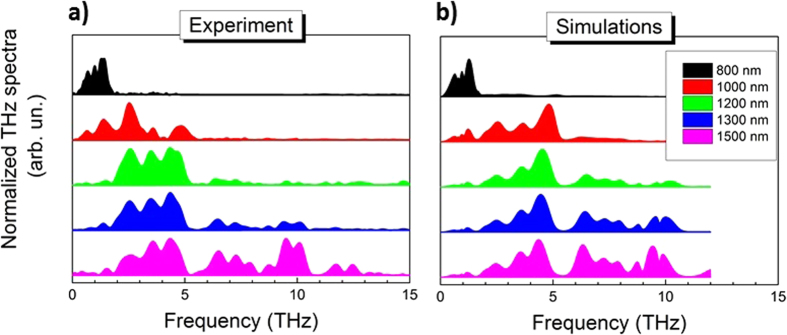
Shaping of the Terahertz spectral output by tuning the pump laser central wavelength. (**a**) Phase-matching conditions in HMQ-TMS give rise to a pronounced dependence of the THz spectrum as function of the pump laser wavelength which provides user control of the generated THz central frequency and the spectral width. The experimentally recorded multi-octave THz spectra cover the lower THz frequency range (800 nm pump) while the entire THz gap (0.1–15 THz) is subsequently covered for longer pump wavelength (1500 nm). The results are excellently reproduced in (**b**) by the corresponding theoretical modeling in the spectral range up to 12 THz (limited by the measured data on refractive index and absorption). Note that the spectra are plotted on a linear scale.

Our approach is extending now the spectral bandwidth tunability across the entire THz gap (Fig. 4) for intense THz pulses. Strongly varying phase matching results in THz emission in different THz spectral regions, as illustrated in Fig. 4(a). The spectral THz width and central frequency show a monotone increase for longer pump wavelengths. This results in a tunability of the spectral width between 2 and 15 THz and the spectral center of mass between 1 and 7 THz, respectively, by simply varying the pump central wavelength while keeping the pulse duration constant. T-ray radiation at a central frequency of 1 THz is produced by the shortest pump wavelength available from our laser system (λ_p_ = 800 nm). A significant spectral shift and broadening in the THz spectrum is observed by using a pump laser spectrum tuned towards the longest available pump wavelength (λ_p_ = 1500 nm). Remarkably, the produced THz spectrum covers four octaves (0.1–2 THz) for the shortest pump wavelength, and expands to more than seven octaves (0.1–15 THz) for the longest. As shown in Fig. 4(b), the measured THz spectra are excellently reproduced by theoretical modeling (see the Methods section) considering the phase matching in the spectral region up to 12 THz where the refractive index and absorption coefficient for HMQ-TMS could be measured (see Supplementary material section).

**Figure 5:**
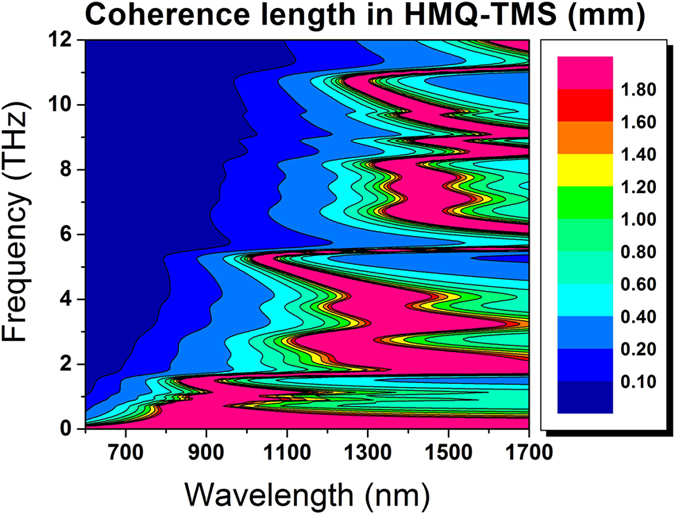
Two-dimensional plot of the coherence length L_c_ = π/Δk for HMQ-TMS gives a direct representation of the optical rectification phase matching mechanism (also known as velocity matching) for different pump wavelengths. The calculations are restricted to frequencies below 12 THz, where the indexes of refraction have been measured. The velocity matching between the pump pulse and the generated THz frequencies depends strongly on the optical wavelengths. In particular, when the pump is shifted toward longer wavelengths large coherence length manifests for broader THz spectrum. In this conditions the velocity matching is achieved over the entire THz gap.

Figure 5 shows the two-dimensional map of the coherence length *L*_*c*_ for HMQ-TMS as function of the pump wavelength λ_p_ and the emitted THz. The coherence length is defined as:


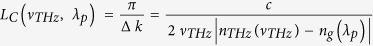


where c is the speed of light in vacuum, Δk is the phase mismatch between THz and pump, n_THz_ and n_g_ are the index of refraction and the group index for the THz frequency, ν_THz_ , and the pump wavelength, λ_p_ respectively (more details on the theoretical modeling are provided in the Supplementary material). The calculations take into account the phase mismatch between the pump and the generated THz radiation upon propagation in the crystal. In Fig. 5 the thickness of the crystal used in the experiment is indicated in bright blue (~0.4 mm). The plot reveals the generated THz spectral feature for the different pump wavelengths available in the experiment. In particular, for the pump wavelength around 800 nm large coherence length (*L*_*c*_ > 1 mm) is obtained only at low THz frequencies. When the pump laser is shifted toward longer wavelengths the coherence length increases for larger THz spectrum and therefore the phase matching is achieved over a broader range covering ultimately the full THz gap. As reported previously^28^ and in the Supplementary material, for HMQ-TMS, the optical group index at wavelength around 1.5 μm (*n*_*g*_ = 2.0) and the THz index of refraction between 1 and 12 THz are close, which gives rise to multi-octave phase matching. At shorter pump wavelengths the index matching occurs only at frequency lower than 2 THz, which results in a narrower THz emission.

**Figure 6:**
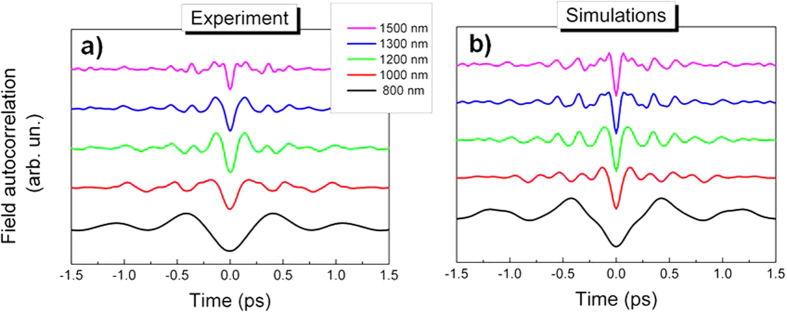
Intense single-cycle Terahertz transients produced by HMQ-TMS (a) THz single-cycle electric field autocorrelation as function of the corresponding pump laser wavelength (same color code as in Fig. 3). The THz pulse duration is tunable between 68 fs (purple line, 1500 nm) and 1100 fs (black line, 800 nm) while the THz single-cycle pulse characteristics are maintained. The theoretically-evaluated temporal field evolutions reported in (**b**) are in excellent agreement with the experimental data.

The bandwidth-adjustable multi octave-spanning THz spectra lead to field transients in the temporal domain whose carrier frequency varies accordingly. For different pump wavelengths the THz pulse duration spans from 1100 fs to 68 fs (Fig. 6). As expected from optical rectification of a 65 fs FWHM pump pulse (constant for all the wavelengths) the THz pulse maintains its transform-limited and single-cycle field shape across the entire temporal tuning range. Theoretical modeling reproduces accurately the waveform shape in the time domain (see Supplementary material).

**Figure 7:**
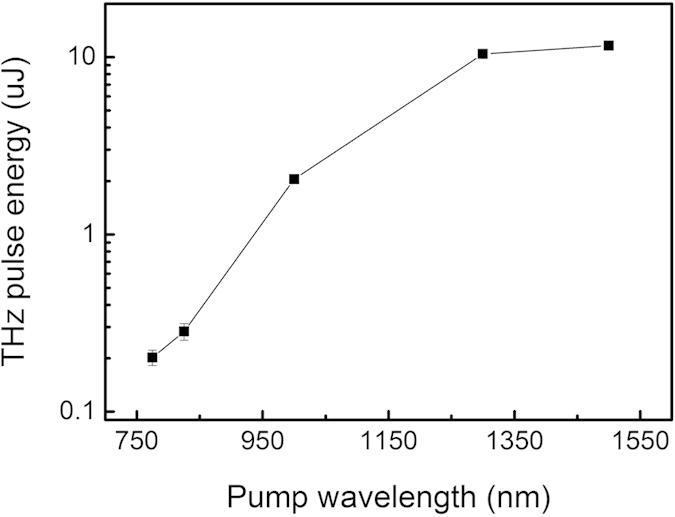
THz pulse energy generated by optical rectification in a small-size HMQ-TMS when pumped at different wavelengths. For direct comparison in all the measurements, the pump fluence is kept at 10 mJ/cm^2^. The results suggest a direct link between the THz energy and the optical rectification phase matching bandwidth. The THz yield increases significantly for pump wavelengths where the emitted spectrum is broader. Upscaling the THz energy is straightforward by employing larger or partitioned HMQ-TMS crystals and by using a stronger pump pulse.

Shown in Fig. 7 is the recorded THz energy as function of the pump wavelength. For all measurements the pump fluence is kept constant at 10 mJ/cm^2^ corresponding to 2 mJ energy for a direct comparison. The results suggest a direct relationship between the THz energy and the optical rectification phase matching bandwidth. The THz yield increases significantly for pump wavelengths where the emitted spectrum is broader. As shown in the plot, pump optical pulse at 800 nm gives rise to a 0.2 μJ THz pulse, while at 1500 nm pump the energy conversion shows a more than 50-fold enhancement.

The corresponding Terahertz transients carry an electric (magnetic) field strength as high as 6.1 MV/cm (2 Tesla) for the pump at 1.5 μm. At shortest pump wavelengths the THz electric and magnetic field still reaches 100 kV/cm, which is in line with theoretical expectations. We recall the reader that the maximum achievable field for lower carrier frequencies is naturally smaller compared to higher frequencies because the diffraction limited spot area and pulse duration scale as 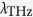
2 and 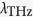, respectively. In contrast to other THz shaping schemes^17^,^18^,^20^,^21^ the single-cycle pulse synthesizer tunable in bandwidth and carrier frequency presented here works across the entire THz gap and at much higher field strengths. Moreover our spectral synthesizer does not rely on lossy dispersive elements or specifically designed optics neither on complex pump laser manipulation which could limit the manageable field strength and bandwidth.

## Conclusions

We demonstrated the production of transform-limited, single-cycle THz pulses with tunable bandwidth and tunable carrier frequency by controlling the exceptional phase-matching conditions in the organic crystal HMQ-TMS via a wavelength-tunable pump pulse. The compact, laser-driven strong-field source covers the entire THz frequency gap between 0.1 and 15 THz by emitting spectrum of variable bandwidth (up to 7 octaves) at high spectral power density. The corresponding THz transients are single-cycle, Fourier-limited and tunable in duration from 68–1100 fs, thanks to a spectral bandwidth tunable between 1 and 15 THz. Presently, our THz pulse shaping concept provides fields up to 6.1 MV/cm (2 Tesla). The scheme is straightforward up-scalable by employing large-size crystals^23^, higher pump power and diffraction limited focusing^24^. The exceptional properties of optical rectification in HMQ-TMS at different pump wavelengths permit spectrum and carrier frequency tuning without the use of lossy dispersive elements. Our simple method represents an important conceptual advance for field-triggered excitation in matter as it allows for the first time to exactly match the stimulus field cycle to the the natural timescale of the investigated physical phenomenon.

## Methods

### THz characterization

For the detection of Terahertz radiation we use a Fourier-transform interferometer based on a first-order autocorrelation which has been cross-checked with an electro-optical sampling (EOS) scheme in GaP and air-biased coherent detection (ABCD)^29^. While the EOS suffers from bandwidth-limitation associated with the detection crystal and probe pulse duration, the autocorrelation and ABCD provides a flat detection response across the full THz gap(0.1–15 THz). The complete reconstruction of the THz electric field is done with ABCD. Due to higher sensitivity the autocorrelation is preferred detection technique for low energy THz while ABCD is applied at highest energy. The Terahertz pulse energy is measured by a calibrated Golay cell and the THz focal spot size is determined by means of an uncooled 2-dimensional micro-bolometer (NEC). The electric field strength is calculated by measuring THz pulse energy, duration and the focal spot size.

### Theoretical Modeling

T-ray yield in HMQ-TMS is evaluated using the theoretical model presented in ref. 30, 31, 32, 33, 34. The calculations, which are described in detail in the Supplementary Material section, take into account velocity matching between optical and THz waves, linear absorption in the optical and THz range, generation crystal thickness and pump pulse duration. The refractive indices and absorption at optical/IR frequencies of HMQ-TMS are taken from ref. 28. The refractive index and absorption at THz frequencies in a broad spectral range (1.2–12 THz) have been measured by THz time-domain spectrometry.

## Additional Information

**How to cite this article**: Vicario, C. *et al.* Intense, carrier frequency and bandwidth tunable quasi single-cycle pulses from an organic emitter covering the Terahertz frequency gap. *Sci. Rep.* **5**, 14394; doi: 10.1038/srep14394 (2015).

## Supplementary Material

Supplementary Information
