# Supplementary material for: Intense, carrier frequency and bandwidth tunable quasi single-cycle pulses from an organic emitter covering the Terahertz frequency gap
Source: Sci Rep. 2015 Sep 24;5:14394. doi: 10.1038/srep14394 (PMC4585874; doi:10.1038/srep14394)
Supplement: Supplementary Information [file srep14394-s1.pdf]

Supplementary material

Intense, carrier frequency and bandwidth tunable quasi single-cycle pulses from an organic emitter covering the Terahertz frequency gap

C. Vicario[1], B. Monoszlai[1], M. Jazbinsek[2], S.-H. Lee[3], O-P. Kwon[3] and C. P. Hauri[1,4*]

[1]Paul Scherrer Institute, SwissFEL, 5232 Villigen PSI, Switzerland

[2]Rainbow Photonics AG, 8048 Zurich, Switzerland

[3]Department of Molecular Science and Technology, Ajou University, Suwon 443-749, Korea

[4]Ecole Polytechnique Fédérale de Lausanne, 1015 Lausanne, Switzerland

* corresponding author: christoph.hauri@psi.ch

Sample preparation

The HMQ-TMS compound is synthesized by a condensation reaction in methanol, which allows a high yield and reduced impurity level compared to metathesis reaction [20, 31]. The condensation reaction with 4-hydroxy-3-methoxybenzaldehyde and 1,2-dimethylquinolinium 2,4,6-trimethylbenzenesulfonate is performed at 70°C. HMQ-TMS crystals are grown by slow-cooling method in methanol with spontaneous nucleation. As-grown HMQ-TMS crystals are cut by a knife along the direction normal to their crystallographic *b*-axis to obtain suitable facet and desired thickness for optical and THz experiments [20].

Air-Biased Coherent Detection (ABCD)

The electric field reconstruction is performed by the air biased coherent detection (ABCD) proposed originally by Dai et al.[29]. While electro-optical sampling suffers from dispersion and phonon absorption in the electro-optical crystal (ZnTe can be used up to 4 THz and GaP up to 9 THz[32], ABCD provides a flat detection response up to very high frequencies. The basic principle of ABCD THz detection is the measurement of the terahertz-field-induced optical second harmonic generated through a third order nonlinear process $\chi^{(3)}$ in air [33]. In the experimental setup the THz pulse and a femtosecond optical probe I_{ω} from the Ti:Sa laser are focused collinearly in the same spot in air. In a reciprocal process of the THz plasma-based generation[11], the THz field E_{THz} produces a second harmonic of the probe pulse $I_{2\omega}^{THz}$ which is proportional to the THz intensity. By measuring $I_{2\omega}^{THz}$ for different probe delay the THz intensity is reconstructed (incoherent detection). When an external AC electric field E_{bias} is applied, an additional second harmonic $I_{2\omega}^{bias}$ is generated. To realize this field in our experimental setup, two electrodes with a 2 mm gap are placed across the

focal spot and polarized with ±2 kV, 25 Hz square voltage synchronized with the laser repetition rate (100 Hz).

When the THz and the bias field are simultaneously applied, the second harmonic signal contains a cross-term at the bias frequency, $I'_{2\omega}$. This quantity is directly proportional to the THz electric field and can be easily isolated with heterodyne detection in a lock-in amplifier. The cross-term is written as:

$$I'_{2\omega}(t) \propto 2[\chi^3 I_\omega(t)]^2 E_{bias} \cdot E_{THz}(t-\tau)$$

We note that $I'_{2\omega}$ is quadratically proportional to χ(3) and $I_\omega(t)$, and linearly proportional to E_{bias} (constant in the experiment) and to E_{THz}. The above equation provides therefore a method for reconstructing the THz electric field evolution by scanning the probe pulse delay and recording $I'_{2\omega}$. Since the detection of the terahertz waves occurs in a nearly nonabsorbing and nondispersive medium (air), the spectrum cutoff is solely limited by the duration of the laser pulse used as a probe. Using short laser pulses, detection bandwidth beyond 120 THz has been demonstrated[34]. In our experimental setup the probe pulse duration (50 fs) corresponds to maximum bandwidth around 20 THz.

Theoretical Modeling

For evaluating the THz-wave generation efficiency by optical rectification in HMQ-TMS we consider the theoretical model presented in Ref [30]. The model takes into account velocity matching between optical and THz waves, linear absorption in the optical and THz range, generation crystal thickness and pump pulse duration. In a non-depleted pump approximation and neglecting cascaded nonlinear optical effects, the electric-field amplitude E_{THz} of the generated THz wave in a nonlinear optical crystal of length L is a function of the pump laser wavelength λ_p and the generated THz frequency ν_{THz} and is given by

$$\left|E_{\mathrm{THz}}(\nu_{\mathrm{THz}},\lambda_p)\right| = \left| \frac{2\pi\mu_0\chi^{(2)}(\nu_{\mathrm{THz}},\lambda_p)\,\nu_{\mathrm{THz}} I(\nu_{\mathrm{THz}})}{n(\lambda_p)\left(\dfrac{c(\alpha_{\mathrm{THz}}(\nu_{\mathrm{THz}})/2+\alpha(\lambda_p))}{2\pi\nu_{\mathrm{THz}}} + i(n_{\mathrm{THz}}(\nu_{\mathrm{THz}})+n(\lambda_p))\right)} \right| L_{\mathrm{gen}}(\nu_{\mathrm{THz}},\lambda_p,L)$$

where c is the speed of light in vacuum, μ_0 the vacuum permeability and $I(\nu_{\mathrm{THz}})$ the Fourier transformed intensity of the pump pulse. Since the nonlinear optical susceptibility coefficient for optical rectification $\chi^{(2)}(\nu_{\mathrm{THz}},\lambda_p)$ is for HMQ-TMS not known yet, we only evaluate relative field strengths as a function of λ_p and ν_{THz}. We also consider the linear optical material parameters: $n(\lambda_p)$ and $\alpha(\lambda_p)$ are respectively the refractive index and absorption coefficient at the pump optical wavelength λ_p, while $n_{\mathrm{THz}}(\nu_{\mathrm{THz}})$ and $\alpha_{\mathrm{THz}}(\nu_{\mathrm{THz}})$ are respectively the refractive index and absorption coefficient at the generated THz frequency ν_{THz}. The important dependence on the crystal length L and the velocity-matching conditions is described by the effective generation length

$$L_{\mathrm{gen}}(\nu_{\mathrm{THz}},\lambda_p,L) = \sqrt{\frac{\exp(-\alpha_{\mathrm{THz}}L)+\exp(-2\alpha L)-2\exp(-(\alpha_{\mathrm{THz}}/2+\alpha)L)\cos(\pi L/L_C(\nu_{\mathrm{THz}},\lambda_p))}{(\alpha_{\mathrm{THz}}/2-\alpha)^2+(\pi/L_C(\nu_{\mathrm{THz}},\lambda_p))^2}}$$

where the coherence length L_C for optical rectification is given by

$$L_C(\nu_{THz},\lambda_p)=\frac{c}{2\,\nu_{\mathrm{THz}}\left|n_{\mathrm{THz}}(\nu_{\mathrm{THz}})-n_g(\lambda_p)\right|}$$

with n_g the group index at the pump optical wavelength.
The refractive indices and absorption $n(\lambda_p)$ and $\alpha(\lambda_p)$ at optical/IR frequencies of HMQ-TMS are taken from Ref [28]. The refractive index and absorption $n_{\mathrm{THz}}(\nu_{\mathrm{THz}})$ and $\alpha_{\mathrm{THz}}(\nu_{\mathrm{THz}})$ at THz frequencies in a broad spectral range (1.2–12 THz) have been measured in this work by THz time-domain spectrometry (see next paragraph).

Refractive index and absorption of HMQ-TMS in a broad THz range

The linear optical properties along the polar axis of HMQ-TMS crystals are measured by using THz time-domain spectroscopy (THz-TDS) in a THz spectrometer *TeraKit* from Rainbow Photonics AG, operating in a broad THz range 1.2–12 THz. The measurements are carried out using 160 µm and 450 µm thick HMQ-TMS crystals cut normal to their *b*-axis and the THz beam polarized along their polar axis. The results are shown in Fig. S1. The refractive index and the absorption data are modeled simultaneously by using a Lorentz 11-oscillator model with peaks occurring at 1.63, 2.13, 2.98, 3.92, 5.47, 6.89, 7.62, 8.34, 8.97, 9.72 and 11.02 THz. The resulting Lorentz curves are considered in the theoretical evaluation of the THz generation efficiency of HMQ-TMS crystals [28,30].

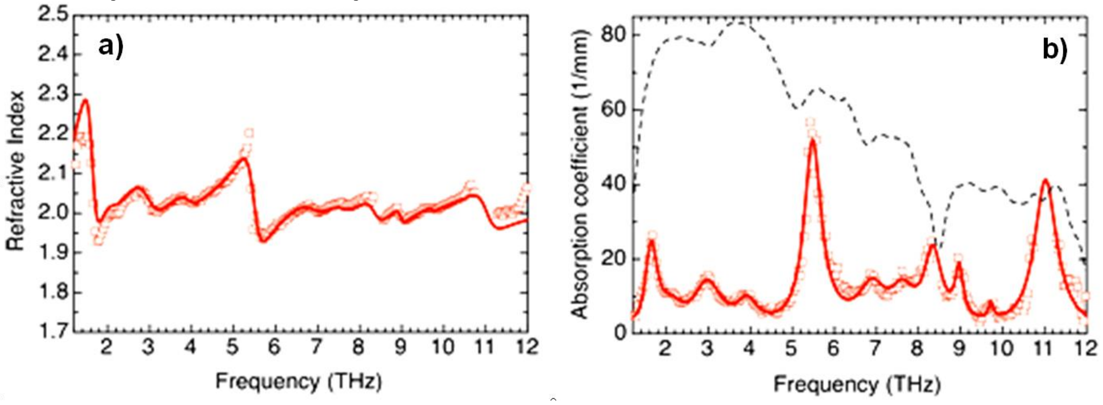

Figure S1. (a) Refractive index and (b) absorption coefficient along the polar axis of HMQ-TMS crystals determined by broadband THz time-domain spectroscopy. Open squares: measured data. Solid curves: best theoretical curve corresponding to a Lorentz 11-oscillator model. The dashed curve in (b) shows the dynamic range of the measurement for a 160 µm thick sample.

Low pass filter transmission

Fig. S2 shows the transmission for the 20 THz low pass filter by QMC instruments used in the experiment. To filter out the residual pump 2 low pass filters have been stacked.

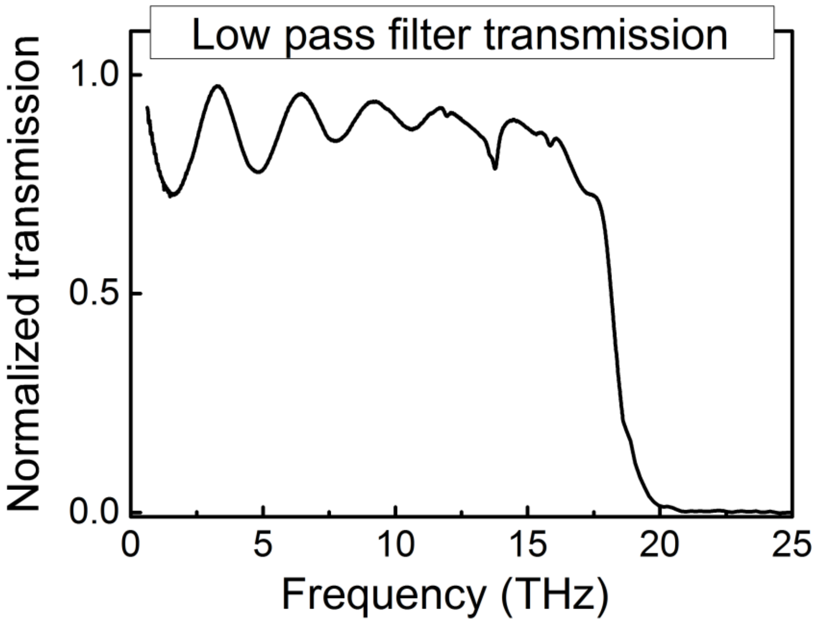

Figure S2. Transmission curve of the 20 THz low pass filter (QMC instruments) used in the experiment.
